# SodSAR: A Tower-Based 1–10 GHz SAR System for Snow, Soil and Vegetation Studies

**DOI:** 10.3390/s20226702

**Published:** 2020-11-23

**Authors:** Jorge Jorge Ruiz, Risto Vehmas, Juha Lemmetyinen, Josu Uusitalo, Janne Lahtinen, Kari Lehtinen, Anna Kontu, Kimmo Rautiainen, Riku Tarvainen, Jouni Pulliainen, Jaan Praks

**Affiliations:** 1 Finnish Meteorological Institute, Erik Palménin Aukio 1, 00560 Helsinki, Finland; juha.lemmetyinen@fmi.fi (J.L.); anna.kontu@fmi.fi (A.K.); kimmo.rautiainen@fmi.fi (K.R.); riku.tarvainen@fmi.fi (R.T.); jouni.pulliainen@fmi.fi (J.P.); 2 Fraunhofer Institute for High Frequency Physics and Radar Techniques FHR, Fraunhoferstraße 20, 53343 Wachtberg, Germany; risto.vehmas@fhr.fraunhofer.de; 3 Harp Technologies Ltd., Tekniikantie 14, 02150 Espoo, Finland; josu.uusitalo@harptechnologies.com (J.U.); janne.lahtinen@harptechnologies.com (J.L.); kari.lehtinen@harptechnologies.com (K.L.); 4 Department of Electronics and Nanoengineering, Aalto University, Maarintie 8, 02150 Espoo, Finland; jaan.praks@aalto.fi

**Keywords:** SodSAR, synthetic aperture radar, InSAR, boreal forest, vector network analyzer, scatterometer, radiometric calibration, temporal coherence

## Abstract

We introduce SodSAR, a fully polarimetric tower-based wide frequency (1–10 GHz) range Synthetic Aperture Radar (SAR) aimed at snow, soil and vegetation studies. The instrument is located in the Arctic Space Centre of the Finnish Meteorological Institute in Sodankylä, Finland. The system is based on a Vector Network Analyzer (VNA)-operated scatterometer mounted on a rail allowing the formation of SAR images, including interferometric pairs separated by a temporal baseline. We present the description of the radar, the applied SAR focusing technique, the radar calibration and measurement stability analysis. Measured stability of the backscattering intensity over a three-month period was observed to be better than 0.5 dB, when measuring a target with a known radar cross section. Deviations of the estimated target range were in the order of a few cm over the same period, indicating also good stability of the measured phase. Interforometric SAR (InSAR) capabilities are also discussed, and as a example, the coherence of subsequent SAR acquisitions over the observed boreal forest stand are analyzed over increasing temporal baselines. The analysis shows good conservation of coherence in particular at L-band, while higher frequencies are susceptible to loss of coherence in particular for dense vegetation. The potential of the instrument for satellite calibration and validation activities is also discussed.

## 1. Introduction

Spaceborne radar remote sensing is particularly useful in Arctic and boreal region remote sensing due to the ability of radar acquiring images without solar illumination and through the clouds. The first synthetic aperture radar, SeaSat [1], opened the era of Arctic remote sensing and started the first time series of Arctic ice. Since the days of Seasat, spaceborne Synthetic Aperture Radar (SAR) has established its operational use in remote sensing of forest, snow, ice, landslides, and many other disciplines.

Despite the wide usage, there still exist challenges which restrict wider the utilization of existing frequent and free data [2]. The major challenge in radar remote sensing is lack of ground validation data, especially validation data acquired by radars in the same frequency band. The interpretation of geophysical parameters from spaceborne radar observations cannot typically solve uncertainties in sensor imaging geometry and calibration, or match the ancillary data with similar temporal resolution.

Ground-based radar remote sensing provides the only solution to this problem. It allows observing radar signatures from natural targets in relatively controlled conditions, and collection of ancillary information with high fidelity. Ground-based radars are therefore the key component in developing and validating physical forward models capable of simulating backscatter intensity and phase signatures [3,4,5], as well as to formulate retrieval algorithms to estimate different geophysical parameters from, e.g., SAR imagery [6]. Performing measurements at, or close to, ground level simplifies the collection of ancillary data, and allows to negate atmospheric and ionospheric effects from the observations. Furthermore, ground-based instruments can typically provide a higher temporal resolution compared to airborne or spaceborne instruments, which allows investigating the effects of rapidly changing environmental conditions (e.g., wind, precipitation) on the remote sensing signal [7]. However, ground-based systems, specially tower-based ones, present an important limitation as they are limited to a much smaller area coverage once they are deployed. They are also susceptible to suffer from environmental harsh conditions, which could cause malfunction.

Several ground-based radar remote sensing instruments have been developed to address the above-mentioned limitations of airborne and spaceborne systems. Several Vector Network Analyzer (VNA)-based instruments have shown to be suitable for ground remote sensing [8,9]. SnowScat [10], a fully polarimetric X-band to K-band radar, has been used to make continuous time series measurements of the radar cross-section of snow [11]. Besides being operated in a static scatterometer setup with a scanning antenna, SnowScat has also been used in tomographic SAR imaging [12]. SnowScat tomographic imaging refers to forming a synthetic aperture in elevation, allowing high resolution studies of the vertical structure of, e.g., snow, ice, or vegetation. SnowScat has also been used for Snow Water Equivalent (SWE) retrieval using Differential Interferometric SAR (D-InSAR) with a multi-frequency approach to reconstruct lost phase cycles [13]. BorealScat [14], which uses P-, L- and C-band, is another fully polarimetric system capable of tomographic imaging. It is based on a VNA and a static 20-element vertical antenna array installed in a tower. The array structure enables simultaneous measurements of the reflected signals and thus avoids temporal decorrelation. BorealScat has been used for temporal surveying of the backscatter from the boreal forest, including the effect of temperature and wind and an analysis of the temporal decorrelation [7]. TropiScat, a similar tower-mounted VNA-based tomographic imaging system, has been used for studying vegetation structure of the tropical forest in [15] at P- and L-band. Moreover, TropiScat-2 extends the capabilities of its predecessor by adding C-band [16]. Furthermore, a tower-based scatterometer AfriScat, operating at P-band, has been used for temporal coherence analysis [17].

To extend the existing test infrastructure beyond the state-of-the-art, we introduce a new, four-polarization (VV, VH, HV, HH) frequency-stepping radar sensor operating between 1 and 10 GHz. The radar has been developed around a commercial VNA by adding a Radio Frequency (RF) front-end that provides signal amplification, antenna switches and an internal calibration path through directional couplers. The VNA and RF front-end are enclosed in a thermally stabilized casing allowing operation in a cold environment under harsh weather conditions. The radar can be operated both in scatterometry mode and imaging SAR-mode; a three-axis pointing device allows directing the radar beam, while SAR imaging capability is achieved by moving the sensor along a 5 m-long horizontal rail. In SAR mode, a time-domain back-projection algorithm is applied to reconstruct the SAR image. Absolute calibration and monitoring of instrument stability is achieved by a combination of internal calibration and external calibration targets.

**Figure 1:**
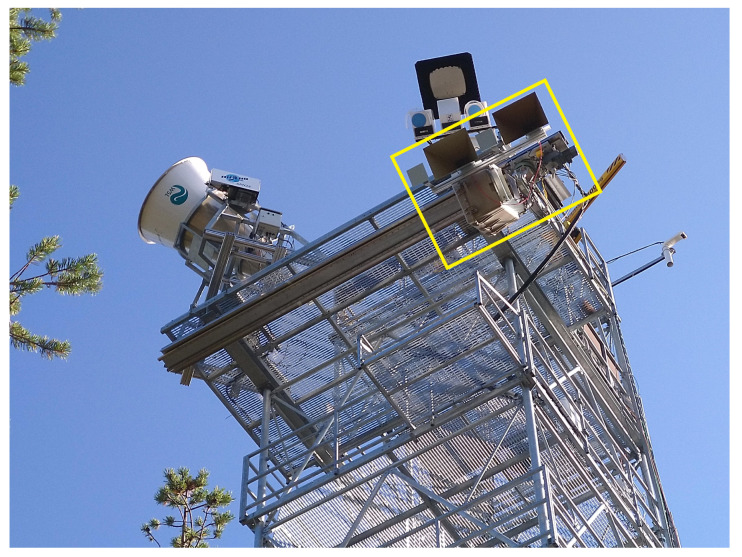
SodSAR mounted on a 21 m tall tower. The radar is mounted on a rail below the platform. Other visible instruments include two passive microwave radiometer systems.

The radar, called SodSAR (Sodankylä Synthetic Aperture Radar) is currently installed on a 21 m high platform overlooking a boreal forest site in Sodankylä, Finland, at the Arctic Space Centre of the Finnish Meteorological Institute. A photograph of the radar system from a low angle perspective is depicted in Figure 1.

The main purpose of the radar is to investigate the behaviour of the backscatter intensity and phase from the boreal forest in varying environmental conditions with good temporal resolution. Furthermore, this is done in an area with a large amount of ancillary data, at L-, C-, and X-band, which are relevant for both operational and planned satellite sensors such as ALOS-2, ALOS-4, ROSE-L (Radar Observing System for Europe), Sentinel-1 or TerraSAR-X. Specific applications include the derivation of forest biomass and the possibility to measure surface parameters such as properties of snow cover and soil through the forest canopy. Of interest are also factors affecting the loss of coherence over forests in temporal interferometric imaging (repeat-pass interferometry). The Arctic Space Centre supports the measurement setup with extensive ground measurement equipment and competent staff to carry out long term experiments.

The paper is organized in the following manner. Section 2 of this paper presents the radar sensor description. The focusing algorithm applied in the SAR imaging mode is presented in Section 3, while the calibration and system stability analysis is presented in Section 4. Section 5 provides an analysis of the temporal coherence of a time series of interferometric, repeat pass SAR images acquired using the sensor over the current test site. Section 6 provides an outlook and discusses potential scientific use of the radar in detail.

## 2. SodSAR Description

This section provides discussion regarding the main aspects of SodSAR. First, thermo-mechanical and electrical design of the radar is presented, describing the systems and subsystems that conform the instrument. Then, considerations about the installation and the operation of the system are presented. The section ends with a table summarizing SodSAR specifications.

### 2.1. Description of Scatterometer Unit

SodSAR as a scatterometer is a fully polarimetric, Stepped-Frequency Continuous Wave (SFCW) radar, especially built for cold climate use. The scatterometer system consists of a Scatterometer Unit and a few auxiliary units: antennas, Power Supply Unit (PSU), control PC and a positioner. The PSU is located on the tower at the same level as the radar and connected to the 230 VAC mains line. It is connected to the Scatterometer Unit (on the platform) via cable and provides its primary voltage, approx. +25 VDC. The control PC is used to read telemetry data and to control the VNA (frequency sweep, output power), the state of the internal RF switches, pointing, and motion.

**Figure 2:**
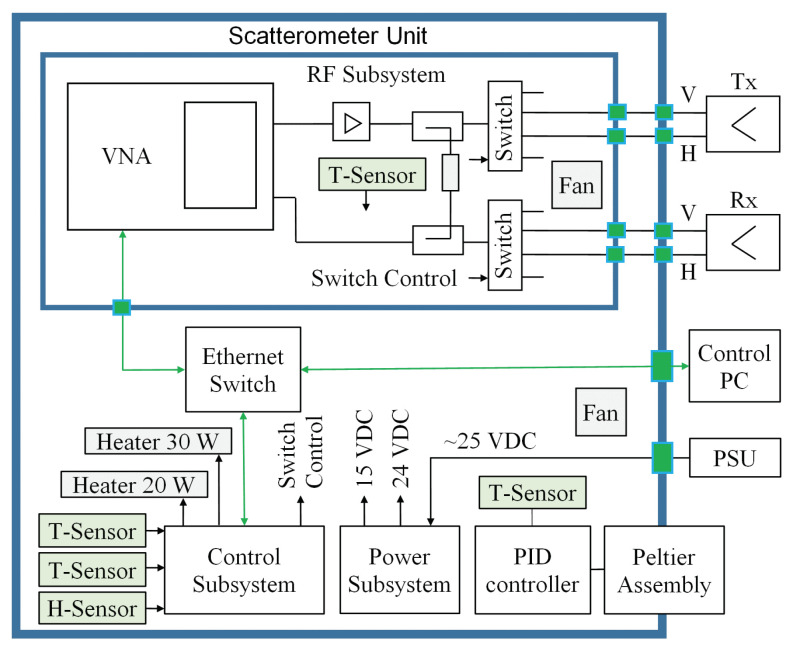
Block diagram of SodSAR’s scatterometer system. Only one antenna pair is shown for clarity. T-sensor and H-sensor stand for temperature and humidity sensor, respectively.

**Figure 3:**
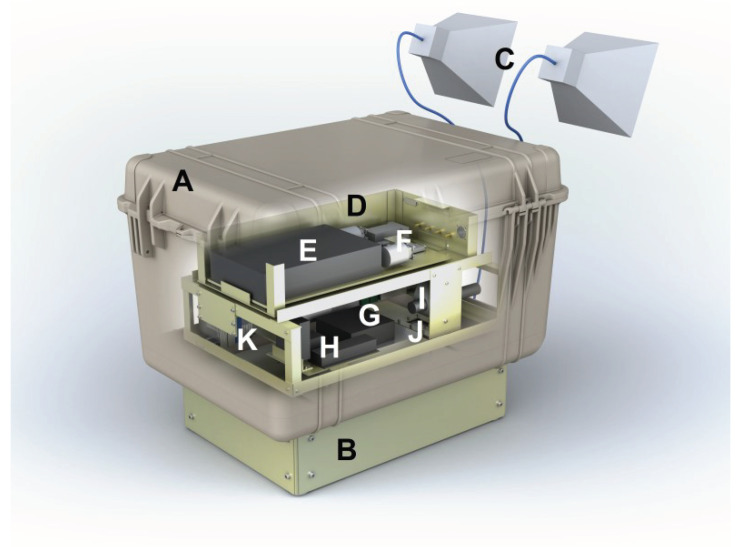
3D illustration of SodSAR’s Scatterometer Unit and antennas: (A) Weather protective casing, (B) pedestal, (C) antennas (only one pair shown), (D) internal enclosure (two side walls and lid not shown), (E) VNA, (F) RF front-end, (G) Peltier-assembly, (H) heating resistors, (I) humidity sensor, (J) connector panel, and (K) Ethernet switch. Control Subsystem and power subsystem are not visible.

The thermo-mechanical design of SodSAR’s Scatterometer Unit follows a “box-in-a-box” principle for advanced thermal control. The outer box (the casing of the Scatterometer Unit) is made of a commercial, water-tight polypropylene transport container. The outer box provides mechanical protection and thermal insulation as well as protection against precipitation and other weather conditions. The outer box is mounted on a pedestal and the overall dimensions of the Scatterometer Unit are approx. 82 × 60 × 61 cm (L × W × H). The inner box (made of aluminium) incorporates the RF Unit that consists of the VNA and all RF components except antennas and external RF cable harness. The other main subsystems incorporated in the Scatterometer Unit are the Control Subsystem, Power Subsystem, Temperature Control Subsystem, and Ethernet Switch. A block diagram of SodSAR scatterometer system is presented in Figure 2 and an illustration of the 3D structure in Figure 3.

The heart of the RF subsystem is a handheld VNA, type Keysight FieldFox N9918A. It is used to measure the $S_{21}$ parameter of the measurement path (amplitude and phase). In the transmit chain, the VNA signal is fed through an RF amplifier, directional coupler, RF switch and an auxiliary antenna. The receive chain is similar to the transmit chain with the exception that it does not include an RF amplifier. The amplifier has approx. 30 dB of gain to boost the VNA signal. The directional couplers in both transmit and receive chains and an attenuator in between form an internal calibration loop. Using this calibration loop the data can be calibrated in post-processing, after transforming the data into time domain. Mutual coupling between transmit and receive antennas provides an additional calibration path. In the time domain analysis (post-processing), antenna coupling, calibration path signal, and ground reflection can be distinguished from each other. Between the couplers and antennas, SP4T switches are included both in transmit and receive chains to select between the two antenna pairs (i.e., the frequency range) and the antenna ports (i.e., the polarization). Two pairs of antennas are currently used: single-polarization horn antennas for 1–2 GHz and dual-polarization horn antennas for 1–10 GHz. The dual polarization antennas can cover L-band at the cost of a wide beamwidth.

Since SodSAR needs to withstand the wide annual temperature variations in Sodankylä (all-time extreme temperatures recorded are −49.5 ${}^{{}^{\circ}}$C and +31.8 ${}^{{}^{\circ}}$C) as well as large diurnal variations, an effective temperature control and stabilization system is needed. First, light colour was selected for the casing to reduce heating by the Sun. Furthermore, the outer box is lined inside with insulation material (extruded polyethylene). In addition, the inner box shields the RF parts from any rapid temperature variations within the outer box. The boxes and their insulation thus provide the first, passive thermal control means. Active temperature control is performed by the Temperature Control Subsystem including a 150 W Peltier assembly (thermoelectric assembly) integrated into the bottom of the outer box. It can both cool and heat the Scatterometer Unit, as needed, and it is independently controlled by a PID controller to keep the inside temperature stable. Finally, two additional heater resistors (20 W and 30 W) are mounted inside the outer box. One or both of these heaters are automatically powered on by the Control Subsystem in extremely low temperatures if the internal temperature drops in spite of Peltier heating. Fans are used to circulate the internal air, to generate a uniform temperature and to cool the power dissipating modules. Within the inner box (RF subsystem), there is also a fan for thermal uniformity and for cooling of the heat- dissipating elements, especially the VNA and the amplifier.

### 2.2. Installation and Operation

**Figure 4:**
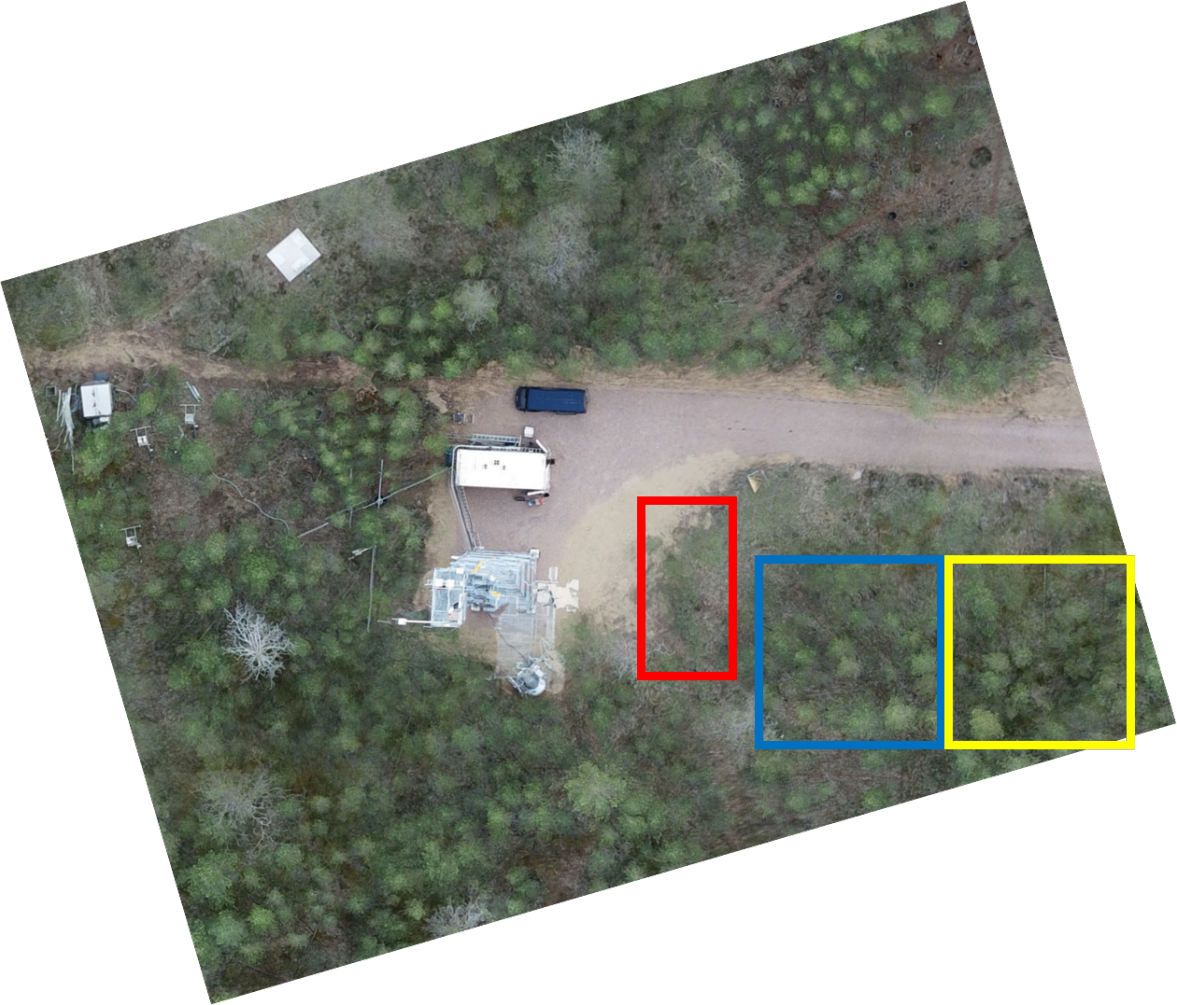
SodSAR scene from nadir, optical image has been captured by a drone during summer 2019.

The SodSAR system has been mounted on a tower overlooking a sparse scots pine (*pinus sylvestris*) forest, at an elevation of approximately 19 m, allowing observations from above the forest canopy (canopy crown height at the site is at approximately 12–15 m). SodSAR is mounted on a 5 m rail enabling movement in the lateral direction for SAR imaging, and equipped with a three-axis pointing device, allowing directing the beam in azimuth and elevation. Positioning along the rail (azimuth) has a precision of millimeters and the pointing device offers sub-degree precision. Movement along the rail is achieved by a rack driven carriage. Figure 4 depicts the area and the tower. The image was taken during the summer of 2019 by a drone. We have chosen three sections from the SodSAR scene analysis, each of them presenting a different composition: Section 1 (red box) covers a patch of open ground, Section 2 (blue box) covers an area with light vegetation and Section 3 (yellow box) covers an area with a lower incidence angle thus the signal is affected more by the surrounding canopy. Section 1 is 9 m long and 6 m width while Sections 2 and 3 are 10 × 10 m. The biomass of each section was estimated based on [18], the required forestry measurements were done during the summer of 2019. Section 1 presents a biomass concentration of 0.63 $\mathrm{kg}/m^{2}$, Section 2 presents 10.30 $\mathrm{kg}/m^{2}$ and Section 3 presents 7.17 $\mathrm{kg}/m^{2}$.

The control PC makes use of dedicated software to automatize the operation of the radar. The software allows selection of the polarization, the start and end frequencies of the VNA sweep, the number of frequency points in the sweep, output power of the VNA, position of the radar over the rail (both in elevation and azimuth) and the starting time. Being able to configure the frequency intervals allows separating the full bandwidth (1–10 GHz) into separate bands. Measurement duration, both frequency sweep time and travel time along the rail, play an important role in SAR, especially for changing scenes. SodSAR is capable of completing a 1 GHz sweep in 4 s and travel the whole rail in around 2 min and 30 s in idle state. The time for a complete measurement depends on its configuration (selected polarization, bandwidth, number of points along the rail…). Nevertheless, the impact of the travel time along the rail can be reduced by selecting a subset of the measurement for later SAR reconstruction. The above-described capabilities suggest a potential for relatively short temporal resolution. However, SodSAR shares the infrastructure with other microwave remote sensing instruments that are exposed to RFI during SodSAR operation. Thus, a compromise between SodSAR acquisition frequency and these other instruments must be met.

In Table 1, a summary of SodSAR specifications is presented.

## 3. SAR Signal Processing

This section describes the necessary signal processing steps to obtain high resolution SAR images from the VNA measurements. First, the sampling requirements affecting the selection of SodSAR operation parameters are analyzed. Then, considerations regarding image resolution are discussed. Finally, the SAR imaging algorithm is described and the section is concluded with an example SAR image.

### 3.1. Sampling Requirements for SAR Operation

To obtain a high spatial resolution in range (radial distance from the radar), the radar needs to transmit a pulse of a short time duration. Alternatively, a large bandwidth corresponding to a short pulsewidth can be synthesized by transmitting a frequency modulated signal. Applying a matched filter for the received modulated waveform will then produce a high range resolution. SodSAR is operated in a SFCW mode to obtain a large frequency bandwidth for the signal. During a frequency sweep, the system sequentially transmits a discrete set of equally spaced frequencies. For each CW frequency segment, the signal is down-converted and averaged to obtain a single IQ sample.

**Figure 5:**
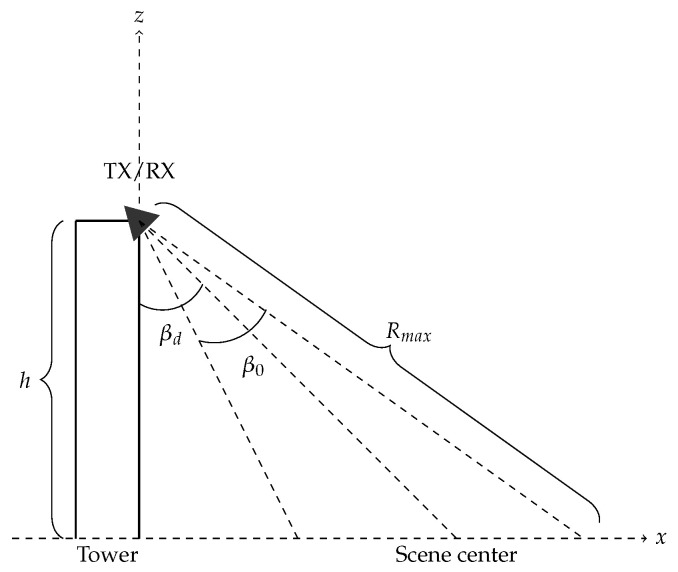
SodSAR imaging geometry from a side-view perspective.

The spacing between the transmitted frequencies determines the sample support of the observable unambiguous time delays. Namely, the maximum unambiguous time delay $T_{max}=1/\Delta f$ for a frequency spacing of Δf. Thus, the maximum unambiguous range is:

(1) $$R_{max}=\frac{c}{2\Delta f},$$

where *c* is the speed of light. Reflections coming from ranges beyond $R_{max}$ will be aliased onto the interval $\left[0,R_{max}\right]$. Designing a suitable frequency step for the measurements depends on the imaging geometry, namely the look angle $\beta_{d}$. The look angle and the antenna radiation pattern (the null-to-null beamwidth $\beta_{0}$) in elevation determine the illuminated (and imaged) area on the ground (see Figure 5). The sample spacing needs to be chosen so that reflections coming from the furthest range $R_{max}$ of the illuminated area remain unambiguous. The bandwidth of the transmitted signal is B=(N−1)Δf, when *N* frequency steps are used. Combining this with (1), we get

(2) $$N=\lceil \frac{2BR_{max}}{c}\rceil +1$$

for the required number of frequency steps.

Another important parameter in SAR operation is the sample spacing along the synthetic aperture. This sample spacing has to be chosen so that the phase change Δϕ of the reflected signal from one aperture position to the next remains unambiguous (Δϕ<2π). The phase change of the reflected signal between two aperture positions can be expressed as

(3) $$\Delta \phi =\frac{4\pi \Delta R}{\lambda },$$

where ΔR is the change in radial distance between the radar antenna and the scatterer in the illuminated area. The range change is ΔR=2Δysinθ, where θ is the off-broadside angle of the scatterer. Thus, to keep the phase changes inside the main lobe of the antenna unambiguous we need to have

(4) $$\Delta y\leq \frac{\lambda }{4\sin\frac{\theta_{0}}{2}},$$

where $\theta_{0}$ is the null-to-null width of the antenna pattern in azimuth (which is twice the maximum possible off-broadside angle of any target inside the main lobe). Since SodSAR is equipped with a wide-band antenna, the beamwidth is frequency-dependent. Thus, to determine the sample spacing Δy, the minimum value of (4) as a function of λ needs to be used. This ensures unambiguous measurements of the illuminated main lobe area for all frequencies. Since the length *L* of the synthetic aperture is fixed by the length of the rail, the number of aperture positions for a SAR measurement will be M=⌈L/Δy⌉+1.

### 3.2. Image Resolution

**Figure 6:**
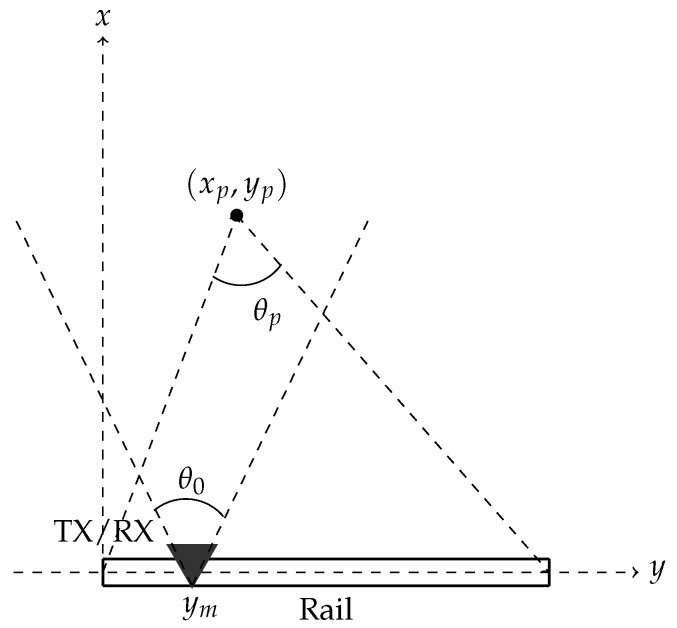
SodSAR imaging geometry from a top-view perspective.

The range resolution of the radar is dictated by the bandwidth *B* of the signal. The ground range resolution δx is additionally affected by the imaging geometry, namely the look angle. It can be expressed as

(5) $$\delta x=\frac{c}{2B\sin\beta },$$

where $\beta =\tan^{-1}\left(\frac{x}{h}\right)$ is the ground range dependent look angle. The cross-range resolution δy is determined by the length of the synthetic aperture and the ground range position. It is obtained as

(6) $$\delta y=\frac{\lambda }{4\sin\frac{\theta_{SAR}}{2}},$$

where $\theta_{SAR}=\min\left(\theta_{0},\theta_{p}\right)$ is the angular size of the synthetic aperture (see Figure 6). The cross-range resolution in (6) depends on ground range, because the angular change $\theta_{p}$ can be smaller than the antenna beamwidth $\theta_{0}$. This is due to the near field imaging geometry and limited rail length, contrasting the more conventional SAR case where cross-range resolution is ground range-independent and depends only on $\theta_{0}$.

### 3.3. SAR Image Reconstruction

To obtain a high resolution in cross-range (azimuth), the SAR principle is utilized. Namely, first, the radar performs measurements in different spatial positions along a so-called synthetic aperture. In order to form the synthetic aperture, the antenna installation is moved along a 5 m rail, perpendicular to range direction. After collection of samples along the rail, the measurements can be treated as if they were performed by a phased array; they are coherently summed with appropriate phase shifts to form the narrow receive beams in each spatial location of the scene (image pixel). This procedure can be repeated for each spatial location (x,y,z) of interest, and the resulting A(x,y,z) is the complex SAR image of the scene. Mathematically, the SAR processing can be implemented as

(7) $$A\left(x,y,z\right)=\sum_{m=0}^{M-1}\sum_{n=0}^{N-1}a_{m}\left(x,y,z\right)S_{mn}\exp\left[-i\frac{4\pi f_{n}R_{m}\left(x,y,z\right)}{c}\right],$$

where $S_{mn}$ is the complex sample at the frequency *n* of the VNA measurement from the aperture position *m*, $R_{m}\left(x,y,z\right)=\sqrt{x^{2}+{\left(y-y_{m}\right)}^{2}+z^{2}}$, $a_{m}\left(x,y,z\right)=1/R_{m}^{2}$ is an amplitude compensation factor due to free space propagation, and $y_{m}$ is the position of the radar along the track. SAR image reconstruction using (7) is equivalent to performing a matched filter for each image pixel [19]. Although evaluating (7) is computationally intensive, we avoid introducing any approximations to speed up the computation: reconstruction accuracy is more important than real-time processing.

**Figure 7:**
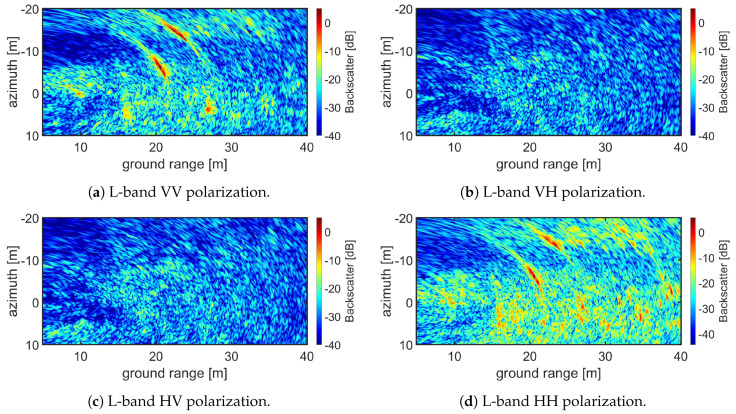
Example of SAR image reconstruction for a full polarimetric acquisition. (**a**–**d**) show the focused images for VV, VH, HV and HH respectively for a L-band acquisition.

An example of a reconstructed SAR image is presented in Figure 7. The image acquisition time was 23 September 2019-06:51:28 UTC. The SAR image has been formed out of M=499 measurements along the track (every 10 cm), each of them consisting of N=501 frequency samples between 1 and 2 GHz with the VNA output power set at −10 dBm, for the full polarimetric case. The maximum range is 75 m. The image covers from 5 to 40 m in ground range and from −20 to 10 m in azimuth. Best range resolution correspond to a point located in the far-end of the image, with value Δx=0.1677 m. Azimuth resolution has been calculated for a point located in the middle of the aperture in near-end of the image, for this point Δy=0.1118 m. Range and azimuth pixel resolution is 0.1 m. In scene two dihedral Corner Reflectors (CR) are present. One located 20 m in ground range and 7 m in negative azimuth and the other located 23 m in ground range and 14 m in negative azimuth. Ideally, dihedral CR only produce a co-polar response and therefore are only seen in VV and HH images, corresponding to Figure 7a,d, respectively.

## 4. Radar Calibration and Measurement Stability

This section describes the radar calibration and the methodology used to check the measurement stability. First, the method for co-polarization and cross-polarization calibration is presented. Finally, we present an analysis of both the backscatter and the phase demonstrating the measurement stability of the radar.

### 4.1. Calibration

The system is calibrated by using calibration targets. To perform radiometric calibration, two calibration targets are placed in the scene, one trihedral CR with an edge length of 90 cm for co-polarization and one tilted dihedral CR with squared faces of 30 cm for cross-polarization. The targets are located in clear line of sight from the radar on its full aperture.

As proposed in [20], the calibration factor can be obtained by integrating over a sufficiently large area containing the response of the calibration target plus the clutter contribution of the background. Then, the background contribution is estimated by integrating over an area close to the calibration target that does not include it:

(8) $$\int \int_{A}E\left\{P\left(x,y\right)\right\}dxdy≃K_{s}\sigma_{pq}^{T}+\int \int_{A}K_{n}\sigma_{n}\left(x,y\right)dxdy,$$

where E{P(x,y)} is the image power, $\sigma_{pq}^{T}$ is the known Radar Cross Section (RCS) from the calibration target, $K_{n}\sigma_{n}\left(x,y\right)$ is the background contribution and $K_{s}$ is the overall system gain. The contribution from the background is now subtracted from the response of the calibration target leaving the term $K_{s}\sigma_{pq}^{T}$ to be solved:

(9) $$\int \int_{A}E\left\{P\left(x,y\right)\right\}dxdy-\int \int_{A}K_{n}\sigma_{n}\left(x,y\right)dxdy≃K_{s}\sigma_{pq}^{T}.$$

The only remaining term to obtain the calibration factor is $\sigma_{pq}^{T}$. The RCS for both targets is calculated from the theoretical value. The RCS of the trihedral CR is:

(10) $$\sigma_{pq}^{tri}=\frac{4\pi a^{4}}{3\lambda^{2}},$$

where *a* is the length of the side edges. A dihedral CR presents pure cross-pol response when a linear polarized incidence wave form an angle of 45${}^{{}^{\circ}}$ with respect to the line of intersection of the two plates that form the structure [21]. The RCS of the dihedral CR is:

(11) $$\sigma_{pq}^{di}=\frac{8\pi a^{2}b^{2}}{\lambda^{2}},$$

where *a* and *b* are the lengths of the dihedral sides.

### 4.2. Measurement Stability

System stability analysis, which was carried out for both backscatter and phase, aims to ensure data reliability. The backscatter stability and phase stability were analyzed by using two separate methods. The analysis was performed in summer conditions.

**Figure 8:**
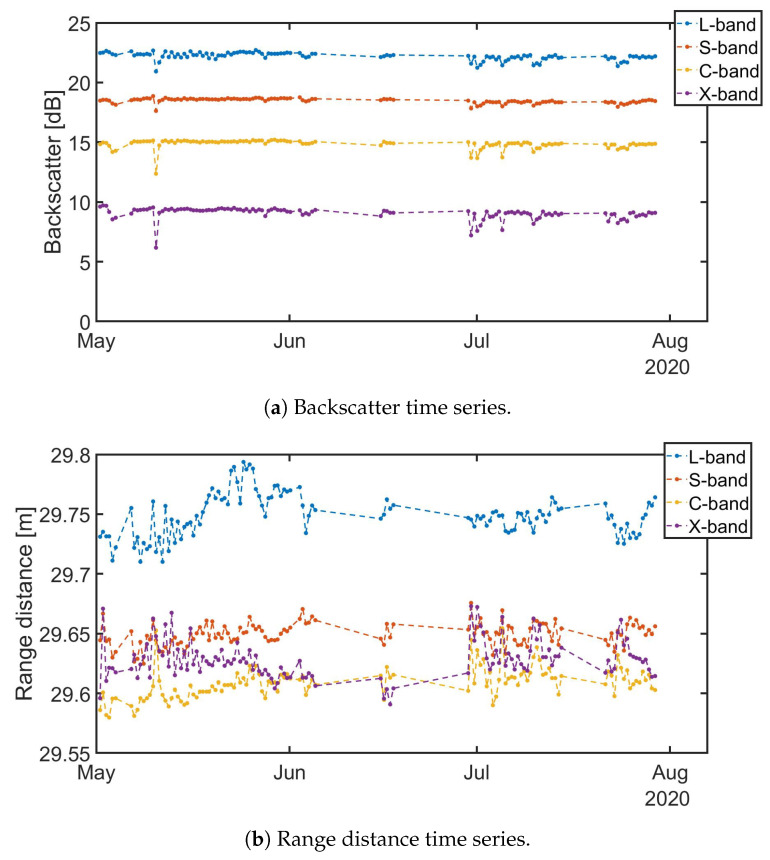
Backscatter and Range distance time series from 1 May 2020 to 29 July 2020, for a total of 117 acquisitions, VV polarization and L-, S-, C- and X-band.

The backscatter stability means that the response of the CR located in clear line of sight remains steady over a period of time. The backscatter for each image is calculated by integrating over an 11 × 11 pixel window over the CR location in the un-calibrated image. Then the average backscatter and the standard deviation is calculated. Furthermore, after analyzing the environmental conditions during the acquisitions a relation between precipitation and deviation were found. This could be due to moisture on the antennas and, therefore, those acquisitions have been left out from the backscatter stability analysis. Table 2 presents the backscatter stability results for three months. Results of the analysis show deviation less than 0.5 dB at all investigated bands. In Figure 8a the time series for L-, S-, C- and X-band are depicted. From the time series we can observe that the deviations from the “nominal” value are localized.

The phase in SAR systems is affected by propagation disturbances (PDs) caused by atmospheric disturbances and uncompensated platform motion. One particular effect of PDs is that they can cause errors in distance measurement [22]. Assessing phase stability using CRs in this particular scenario presents some challenges. While dry snow covering the CR increases the optical path of the signal [13], wet snow over the CRs can mask them due to its low backscatter coefficient [23]. To partially avoid this problem, the CR’s open face was covered by a plexiglass surface, avoiding snow accumulation inside it. In addition, snow accumulation was removed from the CR’s plexiglass surface on a weekly basis during wintertime.

To evaluate the system phase stability the mean range distance from the radar to the CR over the whole sweep was calculated. Figure 8b presents the average range distance from the tower to a CR located in clear line of view. In Table 3 the average range distance for each of the four bands and the standard deviation of the measurements is presented. A total of 117 complete sweeps acquired between 1 May 2020 and 29 July 2020 for L-, S-, C- and X-band have been analyzed. Observed values suggest that for low frequency bands such as L- and S-band, the observed standard deviation is small with respect to the wavelength. Nevertheless, for C- and X-band, the observed standard deviation is significant with respect to the wavelength. This is, of course, a relevant issue for applications that make use of the information from the phase. For those applications, phase correction should be properly addressed. One possible solution for InSAR applications could be checking that the interferometric phase over the CR is zero. We should also note that typical InSAR applications rely (matching common satellites revisit period) on much smaller temporal baselines than the period analyzed above.

## 5. SodSAR as an InSAR

This section introduces SodSAR capability to form interferograms. First, some basic aspects of InSAR are described. Then, a few examples of InSAR products with increasing temporal baseline are presented. Finally, analysis of temporal coherence for four bands (L-, S-, C- and X-) and different sections is presented.

### 5.1. Processing

Coherence in InSAR is defined as the normalized complex cross-correlation between two SAR images:

(12) $$\gamma =\frac{\langle A_{1}A_{2}^{*}\rangle }{\sqrt{\langle A_{1}A_{1}^{*}\rangle \langle A_{2}A_{2}^{*}\rangle }},$$

where $A_{1}$ and $A_{2}$ are the complex-valued signals from the SAR images, * is the complex conjugate and 〈〉 denotes the mathematical expectation.

In practice and under the assumption of distributed targets coherence is calculated by spatially averaging the radar echoes within a moving window [24], such:

(13) $$\gamma =\frac{\sum_{i=1}^{L}A_{1}A_{2}^{*}}{\sqrt{\sum_{i=1}^{L}A_{1}A_{1}^{*}}\sqrt{\sum_{i=1}^{L}A_{2}A_{2}^{*}}},$$

where *i* indicates the *i*th pixel in the coherence estimation window over *L* pixels.

InSAR products suffer from multiple sources of decorrelation, such as temporal, thermal or geometric. Temporal decorrelation is caused by changes in the scenario between acquisitions. Thermal decorrelation is caused by the noise added by the receiver and may be significant especially in areas where the Signal-to-Noise Ratio (SNR) is low, such as the ground. Due to the nature of a tower-based SAR, the spatial baseline is zero, meaning that no geometric decorrelation is present and the focused images can be used to generate interferograms without a Digital Elevation Model (DEM).

### 5.2. InSAR Examples

**Figure 9:**
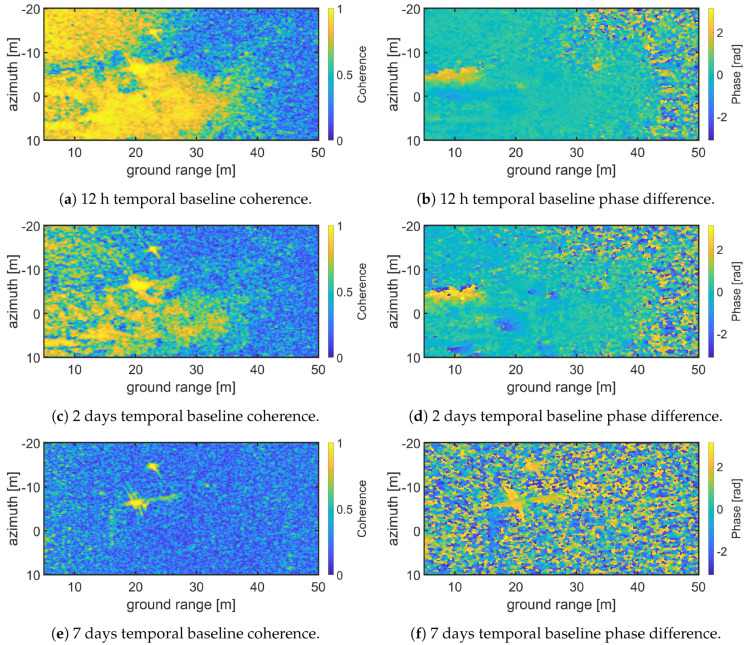
X-band VV polarization interferograms for three different temporal baselines; 12 h (**a**,**b**), 2 days (**c**,**d**) and 7 days (**e**,**f**). Coherence and phase difference are presented for each pair.

In order to demonstrate the capability of SodSAR to measure interferometric pairs separated by a temporal baseline four VV polarization X-band (9-10 GHz) images separated 12 h, 2 days and 7 days were selected. The corresponding acquisition times were: 8 November 2019-14:30:25, 9 November 2019-02:30:25, 10 November 2019-14:30:24 and 15 November 2019-14:30:21 being the first one the master and the rest the slaves images. Each image cover from 5 to 50 m in ground range and from −20 to 10 m in azimuth. The selected coherence window was 5 × 5 pixels. Figure 9 depicts the absolute value of the coherence and the phase for the three interferograms. Figure 9a presents the coherence observed for 12 h temporal baseline. It can be seen that the coherence is conserved in the areas closer to the tower, where less or no vegetation is presented. As the range increases, also the vegetation density increases, producing a loss of coherence at X-band. In areas with high coherence, the phase difference can be recovered. Figure 9b shows the phase difference for 12 h time baseline, it can be seen that it’s close to zero as no motion in the ground occurs between acquisitions. Figure 9b,d show the coherence and phase difference for a temporal baseline of 2 days. Compared to Figure 9a, fewer areas remain coherent. These correspond to less vegetated areas. The phase difference is also close to zero for such areas. Finally Figure 9e,f show the coherence and phase difference for 7 days temporal baseline. In this scenario coherence is almost completely lost and only the CRs remain coherent.

### 5.3. Temporal Coherence Analysis

**Figure 10:**
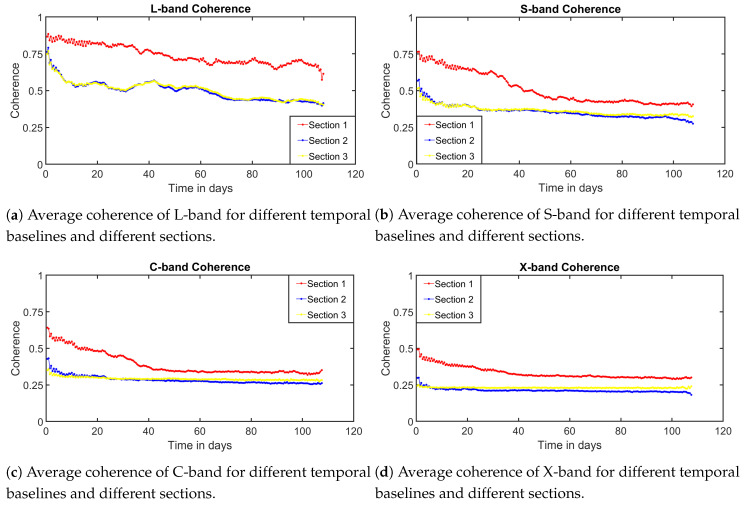
Average observed temporal coherence for four different bands, each of them for three different sections with different characteristics.

The following experiment focuses on analyzing the temporal coherence over the boreal forest, presented in SodSAR’s scene. The dataset consists on 163 acquisitions taken between 6 April 2020 and 29 July 2020. Four frequency bands (L: 1 to 2 GHz; S: 2.5 to 3.5 GHz; C: 5 to 6 GHz and X: 9 to 10 GHz) were measured at VV polarization. A total of N=501 frequency samples were taken along each of the bandwidths, VNA output set to −10 dBm and M=499 samples along the rail. The measurements took place every 12 h with a few maintenance interruptions being the longest between 29 June 2020 and 14 July 2020. The SAR images range from 3 to 50 m in range and form −15 to 15 m in azimuth. InSAR images were generated for every SAR pair with a coherence window of 5 × 5 pixels. For every InSAR image, the mean coherence value of each section was calculated by simply averaging. Finally, for each temporal baseline, individual section coherence values were averaged. Even though the dataset extends up to 114 days, only temporal baselines up to 60 days were analyzed. Other possible sources of decorrelation were not corrected. Figure 10 presents the coherence observed plotted against the temporal baseline for each of the three sections and for the four analyzed bands. Results show strong dependence with frequency, suggesting that higher bands are more susceptible to temporal decorrelation. For Section 1, L-band shows excellent temporal coherence properties, where even extremely long temporal baseline presents good coherence. For higher frequency bands, coherence drops at a faster rate. The explanation for this is that as the wavelength gets smaller, the main backscatter mechanism also does. Small targets, such as leaves, stem or branches, are more susceptible to small movements which cause differences in their response. Similar behaviour regarding frequency can be observed for Section 2 and Section 3. However, coherence drops much faster for vegetated areas, where can be seen that the coherence reaching a ground low value earlier. For all bands, Section 2 was less affected by decorrelation than Section 3, although similar temporal properties are observed. The lack of acquisitions for temporal baselines shorter than 12 h limits the possibility to fully evaluate the results of the experiment as for higher frequency bands coherence drops rapidly.

## 6. Potential Applications of SodSAR

SodSAR gives the possibility for versatile remote sensing investigations. Operating as an interferometric SAR, the instrument will allow exploring in detail the potential to derive the SWE by means of repeat-pass SAR interferometry [25]. This technique essentially derives the increase in SWE between two consecutive SAR images, exploiting a quasi-linear relation between the increase in SWE and the change in radar signal path length induced by refraction at the snow-air interface. The technique was demonstrated at the Sodankylä site using SnowScat [13]. For spaceborne applications, the use of a lower (e.g., L-band) frequency is desirable to avoid phase wrapping from significant snow inclusions. SodSAR allows investigating the applicability of a wide range of frequencies for this purpose. The relatively high temporal resolution of the imaging (12 h) allows to closely investigate the effect of changing weather conditions on the conservation of coherence, which is essential for the proposed interferometric imaging technique. These investigations are relevant for, e.g., the proposed TanDEM-L and ROSE-L missions. Furthermore, exploiting the coherence of interferometric SAR image pairs separated by a physical baseline to derive properties of boreal forests was recently proposed [26]. SodSAR provides the potential to study the applicability of the method over different frequencies and polarizations. Using SodSAR in the present configuration, two physically differing baselines can be achieved by slightly modifying the incidence angle for the consecutive SAR pairs.

## 7. Conclusions

In order to support spaceborne radar remote sensing with high fidelity ground measurements, ground-based radar systems are needed. We presented SodSAR, a fully polarimetric tower-based SAR operating between 1 and 10 GHz. The instrument is intended to collect data from snow, soil and boreal forest in order to characterize their backscatter and phase properties over long time series with good temporal resolution.

SodSAR is based on a commercial VNA allowing SFCW scatterometer measurements. Mounting on a five-meter long rail, movement across it allows measurements over a synthetic aperture for SAR images reconstruction. SodSAR’s Scatterometer Unit is made using a “box-in-a-box” principle. The inner box contains the RF subsystem while the outer box protects the system against weather conditions and includes all other subsystems, such as internal temperature control. Depending on the configuration, certain sampling requirements must be fulfilled for successful SAR operation. We described those requirements and presented the SAR image focusing technique with its mathematical implementation.

The calibration technique was described along with an analysis of the measurements ensuring its reliability. Results showed that both backscatter and phase over permanent targets were stable over the corresponding analysis periods, those being constrained by the particular environmental conditions during the acquisition time.

We also discussed SodSAR capability to form interferograms. InSAR techniques allow observing changes in the phase of the scene. An example of an interferogram with a common master and three different slaves at different temporal baselines was depicted. An analysis of temporal decorrelation properties as a function of the temporal baseline for four bands and three different sections of the SAR images was presented. The results showed the great impact of vegetation and increased effect of frequency in temporal decorrelation.

The potential applications of SodSAR for upcoming years were considered taking into account the possibilities of configuring the measurements by selecting the frequency bands, image resolution and polarizations.

Ground-based radar systems with above-mentioned capacities are not widely available, making SodSAR a unique instrument. SodSAR offers a high degree of flexibility in the measurement setup, allowing band selection, resolution or temporal baseline among other parameters. Furthermore, SodSAR is not only able to generate SAR images, operation in scatterometer mode is also possible.

The present piece of work also encompass a rich description of thermal, mechanical and electrical design considerations, along with its installation and operation.

## Figures and Tables

**Table 1:** SodSAR specifications.

Parameter	Value	Comments
**Frequency Range**	1–10 GHz	
**Transmit Power Range**	From −18 dB to +25 dB	At antenna input
**Number of Frequency** **Measurement Points**	From 3 to 10,001	
**Polarizations**	Linear VV, HH, VH and HV	
**Measurement**	Amplitude and phase of the $S_{21}$ parameter	
**Internal Calibration**	Included	
**Remote Control**	Included	Via control computer
**Nominal Power Consumption**	270 W	
**Worst Case Power Consumption**	520 W	
**PSU Input Voltage**	230 V AC	
**Scatterometer Unit Voltage**	25 V DC	
**Outer Dimensions**	82 × 60 × 61 cm	L × W × H, including feet
**Weight**	Approximately 20 kg	
**Antennas**	External	Enabling adjustable incidence angle
**Antenna Pair 1 Beam Width**	61–66${}^{{}^{\circ}}$ at 1 GHz; 24–25${}^{{}^{\circ}}$ at 10 GHz	Taking into account both orthogonal beam cuts and both polarizations (V and H)
**Antenna Pair 2 Beam Width**	36–33${}^{{}^{\circ}}$ at 1 GHz; 21–19${}^{{}^{\circ}}$ at 2 GHz	Taking into account both orthogonal beam cuts and both polarizations (V and H)
**Positioning Along the Rail**	From 10 to 4990 mm	Margins added for safety
**Precision Along the Rail**	Millimeter Precision	
**Rail Displacement Device**	Rack Driven Carriage	
**Incidence Angle**	0–90${}^{{}^{\circ}}$	
**Azimuth Angle**	−60–60${}^{{}^{\circ}}$	With respect to the perpendicular to the rail
**Pointing Precision**	0.1${}^{{}^{\circ}}$	For both Incidence and Azimuth Angles
**Range Resolution**	0.16–1.62 m	
**Azimuth Resolution**	0.11–0.07 m	

**Table 2:** Average backscatter and standard deviation results from the measurements between 1 May 2020 and 29 July 2020, for a total of 117 acquisitions and VV polarization. The calibration target used was a CR located in clear line of sight from the radar.

Band	Average Backscatter (dB)	Standard Deviation (dB)
L	22.208	0.280
S	18.497	0.161
C	14.902	0.249
X	9.123	0.351

**Table 3:** Average range distance and standard deviation results from the measurements between 1 May 2020 and 29 July 2020, for a total of 117 acquisitions and VV polarization. The calibration target used was a CR located in clear line of sight from the radar.

Band	Average Range Distance (m)	Standard Deviation (m)
L	29.750	0.018
S	29.649	0.009
C	29.609	0.013
X	29.628	0.016
